# Longitudinal exploration of the delivery of care following a successful antenatal practice change intervention

**DOI:** 10.3389/fmed.2025.1476083

**Published:** 2025-05-09

**Authors:** Alix Hall, Emma Doherty, Nicole Nathan, John Wiggers, John Attia, Belinda Tully, Elizabeth J. Elliott, Christopher Oldmeadow, Simon Chiu, Melanie Kingsland

**Affiliations:** ^1^School of Medicine and Public Health, The University of Newcastle, Newcastle, NSW, Australia; ^2^Hunter Medical Research Institute, New Lambton Heights, NSW, Australia; ^3^Hunter New England Population Health, Hunter New England Local Health District, Newcastle, NSW, Australia; ^4^Discipline of Child and Adolescent Health, Faculty of Medicine and Health, The University of Sydney, Sydney, NSW, Australia; ^5^Sydney Children's Hospital Network, Kids Research, Sydney, NSW, Australia; ^6^School of Health Sciences, Faculty of Health and Medicine, and Priority Research Centre for Physical Activity and Nutrition, University of Newcastle, Newcastle, NSW, Australia

**Keywords:** sustainment, sustainability, interrupted time series, segmented regression, implementation

## Abstract

**Introduction:**

This is a secondary analysis of a stepped-wedge trial. The sustainment of evidence-based care is essential for ongoing population benefits. In a randomized stepped-wedge controlled trial of public maternity services across three health sectors in New South Wales, Australia, we demonstrated a positive practice change related to addressing alcohol use during pregnancy. This change followed a 7-month implementation strategy conducted between February 2018 and November 2019. However, evidence suggests that the impact of implementation strategies may reduce over time. It is important to document when and if recommended care reduces, so that timely support for sustainment can be provided.

**Methods:**

As a secondary analysis, an interrupted time series analysis of outcomes from the largest sector of the randomized stepped-wedge controlled trial was conducted. The analysis explored the rate, time points, and extent of change in women’s reported receipt of recommended antenatal care for alcohol consumption, following delivery of an effective implementation strategy.

**Results:**

A total of 4,909 (82% consented) women were surveyed. The proportion of pregnant women receiving recommended care reduced significantly per week following the withdrawal of implementation support, for three of the four outcomes: assessment of alcohol consumption (% change per week: −0.66, 95% CI: −1.1, −0.26); advice not to consume alcohol during pregnancy and of potential risks (% change per week: −0.63, 95% CI: −1.1, −0.22); and complete care relevant to alcohol risk level (advice and referral) (% change per week: −0.64, 95% CI: −1.1,-0.22). Similar results were observed regardless of the timing of antenatal visits. A more rapid decline occurred for most outcomes from the end of implementation until approximately 30 weeks post-implementation.

**Discussion:**

Despite a reduction in the receipt of recommended care, rates were still higher post-implementation than pre-implementation. Receipt of recommended antenatal care for alcohol consumption declined after active implementation support was withdrawn. The findings suggest the need for ongoing monitoring of care delivery and the introduction of additional sustainability strategies at key time points post-withdrawal of implementation support.

## Highlights

- Delivery of evidence-based care often declines following the withdrawal of implementation support.
- An understanding of the rate, time points, and extent of change in care delivery is needed to identify if and when additional sustainment support is required.
- Few studies collect continual data following active implementation to allow for such investigations.
- This secondary analysis explores the rate, time points, and extent of change in delivery of recommended antenatal care addressing alcohol consumption during pregnancy, following delivery of an effective practice change intervention.
- The findings suggest the need for continued monitoring of care delivery and provision of additional sustainment support following the withdrawal of implementation support.

## Introduction

Alcohol consumption during pregnancy carries adverse effects on the pregnant woman and unborn child (1). Clinical practice guidelines (2–4) recommend that healthcare providers (i) routinely assess alcohol consumption by all pregnant women throughout their antenatal care using a validated tool; (ii) advise all women on the potential harms of alcohol consumption during pregnancy and recommend abstinence; and (iii) refer women to support services appropriate to their level of risk to assist in abstaining from alcohol consumption. However, the provision of all three elements of care is less than optimal (5).

In a recent randomized stepped-wedge controlled trial undertaken in public maternity services in three health services in New South Wales, Australia (i.e., sectors), it was found that a multi-strategy practice change intervention (i.e., implementation intervention) was effective in increasing the proportion of women who reported receiving all individual and combined elements of recommended care (assess, advise, and refer) appropriate to their level of alcohol risk (6). To have an impact, an implementation intervention needs to result in the continued delivery of guideline-recommended care long term, beyond the provision of initial implementation support. This is commonly referred to as sustainment (7). Emerging evidence suggests that once initial implementation support or funding is withdrawn, the impact of effective implementation strategies often diminishes (8–10). For example, in a systematic review of trials assessing the sustainment of health professional’s adherence to clinical practice guidelines, only seven of 18 evaluations illustrated 100% sustainment of professional adherence more than 1 year after active implementation (8). There is no agreed definition of what constitutes a successful rate of sustainment (11). However, recent definitions emphasize the need for the target behavior change or practice to be maintained to a level that continues to produce benefits for individuals or the system (10–12).

Although the effects of implementation interventions often reduce over time, little is known about when, how quickly, and to what extent such reductions occur. According to the Dynamic Sustainability Framework, sustainability is a dynamic process that is impacted by the continually changing environment in which the intervention/model of care is being delivered (13). To ensure that sustained delivery is optimized and appropriate support is provided, continuous monitoring, evaluation, and refinement of the intervention and implementation strategies are required (13).

To adequately assess and understand the complex process of sustainability, longitudinal designs (10, 14) and statistical analyses that allow modeling of complex, non-linear relationships are necessary (14). Measurement of sustainment should also be considered from the outset of the implementation process, rather than at the end, when it may be too late to identify when improvements or additional support are needed (13). Due to short funding periods and the difficulties of undertaking continual data collection, long-term assessments are rare (14). Several examples do exist, where data across multiple time points have been used to gain a greater understanding of how implementation effects change over time (15, 16). In one study conducted in the general practice setting in Forth Valley, Scotland, (16) an interrupted time series was used to examine how prescribing rates changed during and following a 12-month quality improvement intervention. An improvement in high-risk non-steroidal anti-inflammatory drugs was found at the end of the intervention period, but also that such effects began to wane in the 12 months following the intervention phase (16). In another example, (15) *post-hoc* analyses of a cluster randomized controlled trial conducted in 20 hospitals across three Australian states, examining thrombolysis rates across time, illustrated initial improvements toward the end and directly after active implementation support, followed by rapid declines thereafter (15). These studies illustrate the advantages of assessing implementation effects across time in terms of understanding the potential for sustainment and if and when additional support and improvements may be needed. There are also examples where the implementation and/or sustainment of alcohol screening and brief behavioral interventions generally (17), and in the context of maternal health specifically (18), have been investigated. While these studies provide important insights into the potential barriers and facilitators of implementing and sustaining these interventions, these studies use predominately qualitative and case study methods, which do not allow for a comprehensive assessment of how implementation rates change over time. We are unaware of similar studies that assess implementation changes over time that address the sustainment of antenatal care addressing alcohol consumption.

**Figure 1:**
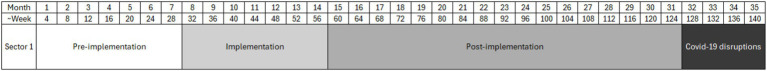
Overview of study phases illustrating the number of months of data collection for each of the phases of pre-implementation, implementation, and post-implementation.

The randomized controlled stepped-wedge trial undertaken by Doherty et al. (6) provides a unique opportunity to undertake a secondary analysis of women’s receipt of guideline-recommended care in relation to alcohol consumption over time. While the primary analysis followed recommended practice for a stepped-wedge design (19), it only informs us whether the level of care received is on average higher across the entire post-implementation period combined, compared to the pre-implementation period combined. It does not examine whether the observed increase in recommended care changes after active implementation support has ended. In this study, many, but not all the practice change strategies, were intended to continue to support the delivery of the recommended model of care following the implementation period, with the primary exceptions being the withdrawal of the clinical champion and educator and the provision of academic detailing and audit and feedback. Thus, an examination of how care continued to be delivered post-active implementation support will provide an opportunity to assess whether implementation effects are being sustained, or if and when additional support may be needed to support long-term sustainment. In this study, the three participating sectors were randomly allocated to one of three wedges. Each wedge started with baseline data collection (or pre-implementation phase) and then moved one by one into receiving the multi-strategy implementation intervention (or implementation phase), which lasted for 7 months. This was followed by a period of post-implementation data collection (or post-implementation phase), which ranged from 9 months for sector three to 21 months for sector one (see Figure 1 for a depiction of sector one’s research phases). The extended follow-up data collection for sector one provides an ideal opportunity to explore the sustainment of this implementation intervention and assess whether care changed during the post-implementation phase following receipt of the implementation intervention.

In a secondary analysis of data collected from public maternity services located in the largest health sector (i.e., a geographically and administratively defined region overseeing healthcare services in that region) from the original stepped wedge trial conducted by Doherty et al. (6), we aimed to:

1. Assess the rate of change in the receipt of care post-implementation for all antenatal visits combined (primary analysis).
2. Assess the rate of change in the receipt of care post-implementation separately by antenatal visit (i.e., initial and subsequent visits).
3. Identify specific points during post-implementation where the rate of change in care received is more rapid.
4. Describe the extent of care receipt at the end of each implementation phase and directly following key time points identified for all antenatal visits combined.

## Methods

### Study design and setting

We conducted an exploratory secondary analysis of data collected during a randomized stepped-wedge controlled trial, which assessed the effect of a multi-strategy practice change intervention on the delivery of recommended antenatal care addressing alcohol consumption by women during pregnancy by healthcare providers (6, 20) (Registration number: ACTRN12617000882325, date registered: 16/06/2017). Human research ethics approval was obtained from the Hunter New England Human Research Ethics Committee (HNELHD: 16/11/16/4.07), the University of Newcastle (H-2017-0032), and the Aboriginal Health and Medical Research Council (1236/16).

All 28 public maternity services within three sectors of the Hunter New England Local Health District of New South Wales, Australia, participated in the trial (conducted between July 2017 and May 2020). For this secondary analysis, only data from sector one were re-analyzed as insufficient data were available from the other two sectors, and sector one had the longest period of data collection for the post-implementation period (~17 months), making it the most suitable for this analysis. Data from all three sectors could not be combined as each sector moved through the phases of implementation at different time points. Data collection for sector one is described in Figure 1. For this secondary analysis, the last 4 months of data were excluded due to unexpected disruptions caused by COVID-19.

### Participants

The implementation intervention was delivered to all public maternity services, comprising 14 antenatal care teams for sector one.

Pregnant women were eligible to complete a study survey if they were between 12 and 37 weeks gestation; were attending the maternity service for either their scheduled first antenatal, 27–28 week gestation, or their 35–36 week gestation antenatal visit in the preceding week; were ≥ 18 years; had a sufficient level of English and were mentally and physically capable of completing the survey; and were receiving the majority of their antenatal care via the public health system. Women who had already given birth, had a negative pregnancy outcome, had completed a survey within the last 4 weeks, or had previously declined participation were ineligible.

Every week, a random sample of 105 eligible women from all three sectors was generated using the appointment system and medical record data; approximately 75% were recruited from sector 1. The percentage of women selected per week was equal for those attending their initial visit (~29%) and 27–28 week gestation visit (~29%). There was a slight oversampling of women attending their 35–36 week gestation visit (~43%) to allow for a higher proportion becoming ineligible due to giving birth. Selected women were sent a study information sheet, and non-Aboriginal women were called 1 week later and invited to complete the survey via computer-assisted telephone interview (CATI) or online. Based on advice received regarding culturally appropriate survey approaches for Australia’s First Nations peoples, women who identified as Aboriginal or Torres Strait Islander origin and/or were attending an Aboriginal Maternal Infant Health Service were sent a text inviting them to complete the survey via CATI or online (6).

### Model of care

Healthcare providers were supported to provide women with the following three elements of guideline-recommended care during their antenatal visits at three time points: initial visit, 27–29 weeks gestation, and 35–37 weeks gestation:

1. *Assessment of alcohol consumption using a validated tool:* Healthcare providers were to use the three-item Alcohol Use Disorders Identification Test-Consumption (AUDIT-C) tool (21, 22) to assess all pregnant women’s alcohol consumption.
2. *Provision of brief advice regarding the potential harms of alcohol consumption during pregnancy and recommend abstinence:* All women were to be advised that it is safest not to consume alcohol during pregnancy and of the potential risks associated with alcohol consumption during pregnancy.
3. *Referral of women to appropriate support services based on their level of alcohol consumption risk:* Women with alcohol consumption classified as medium risk (AUDIT-C score: 3–4) were to be referred to the free government Get Healthy in Pregnancy telephone coaching service (23) with Aboriginal women also offered referral to counseling at Aboriginal Community Controlled Health Services. Women with alcohol consumption classified as high risk (AUDIT-C score: 5 +) were to be referred to the drug and alcohol service provided by the health district.

### Implementation intervention

To support the implementation of this model of care, a multi-strategy practice change intervention was delivered over a 7-month period (between February and August 2018 for sector one). The intervention is described in full in the study protocol (20) and primary outcome paper (6). It was designed to address the key impediments to increasing and sustaining the delivery of the recommended model of care (5). Strategy selection, content, and delivery were informed by current evidence, behavior change experts, practitioner input, and cultural inclusion informed by Aboriginal women, Aboriginal health staff, and local community members and organizations. Broadly, the strategies included leadership and managerial supervision, development of local clinical practice guidelines, electronic prompts and reminders, dedicated clinical champions, provision of educational materials and meetings, academic detailing and audit and feedback, and monitoring and accountability for the performance of the delivery of healthcare. Five of the seven strategies were designed to be integrated within the maternity service’s usual systems and processes, with the intention they would continue as part of routine practice to support the delivery of the recommended model of care following the implementation period and without the external support provided as part of the practice change intervention. Only the dedicated clinical champion and educator, and the provision of academic detailing and audit and feedback strategies were not continued following the implementation period.

### Data collection and outcomes

Data from pregnant women were collected continuously on a weekly basis for the entire 35-month study period (see Figure 1).

### Characteristics of participating women

Women reported their age, Aboriginal and Torres Strait Islander origin, education, employment, marital status, first/subsequent pregnancy status, and the antenatal care providers they saw during their visit in the self-report surveys. Women also completed the AUDIT-C (21, 22) as a measure of their alcohol consumption risk level. Total scores are classified as no risk (0); low risk (1–2); medium risk (3–4); and high risk (5+) (24).

### Receipt of recommended model of care

Women were asked to indicate (yes, no, or do not know) whether they received each of the elements of recommended care during their recent antenatal visit. Specifically, they were asked (i) whether their healthcare provider assessed their alcohol consumption with question/s consistent with the AUDIT-C, (ii) whether they were advised not to consume alcohol during pregnancy and on the potential risks associated with consuming alcohol, and (iii) whether they were offered a referral for further support for abstaining from alcohol consumption during pregnancy.

The primary outcomes were the proportion of women who received (i) assessment via the AUDIT-C; (ii) both components of advice (i.e., advised not to consume alcohol while pregnant and of the potential risks associated with consuming alcohol); (iii) complete care appropriate to alcohol risk level (both components of advice and offer of referral if medium or high risk); and (iv) all components of guideline-recommended care appropriate to alcohol risk level (i.e., assessment and complete care). Consistent with the original trial, all outcomes were assessed across all visits combined (i.e., initial antenatal visit, 27–29 weeks gestation, and 35–37 weeks gestation) (6). However, due to significant differences in the effects observed on outcomes in the later appointments (i.e., 27–29 weeks gestation and 35–37 weeks gestation) (6), we also assessed all outcomes separately for initial and subsequent visits (i.e., 27–29 weeks gestation and 35–37 weeks gestation).

### Statistical analysis

All analyses were conducted in R version 4.1.0 (25). Linear segmented regression models were conducted for each of the outcomes across all visits combined (primary analysis), as well as separately for initial and subsequent visits. Data were assessed for autocorrelation, which was not present. Three segments, one for each of the implementation phases (i.e., pre-implementation, implementation, and post-implementation), were specified in each model. No confounders were included in the models. A break-point analysis for all antenatal visits combined was conducted to estimate additional segments in the post-implementation phase where rates changed more rapidly. The regression coefficient (representing the percentage change in outcomes per week), 95% confidence intervals, and Type III *p*-value are reported. Descriptive statistics were used to describe the levels of care received by women for the last 4 weeks of each implementation phase and following any significant break points identified. An alpha level of 0.05 was used to determine statistical significance.

## Results

### Participants

A total of 9,474 women were sampled over the entire study period, of which 5,996 (63%) were eligible to participate. Of eligible women contacted, 4,927 (82%) consented to complete a survey, with 4,909 (82%) completing a survey. The characteristics of participating women were similar across implementation phases (see Table 1).

**Table 1:** Sector one participant characteristics.

Characteristics	Pre-implementation	Implementation	Post-implementation
Months of data collection	8.5	6.25	16.5
Total number of responses	1,309 (82.6%)	1,028 (81.5%)	2,572 (80.0%)
Participants			
Age			
18- < 25	240 (18%)	138 (13%)	375 (15%)
25- < 35	840 (64%)	687 (67%)	1,634 (64%)
35+	228 (17%)	203 (20%)	563 (22%)
Aboriginal and/or Torres Strait Islander origin	70 (5%)	61 (6%)	117 (5%)
Education level			
Completed high school or less	362 (28%)	285 (28%)	652 (25%)
TAFE^a^ or diploma	481 (37%)	366 (36%)	893 (35%)
University	465 (36%)	377 (37%)	1,026 (40%)
Employment status			
Employed	908 (69%)	755 (73%)	1898 (74%)
Not employed	400 (31%)	272 (26%)	674 (26%)
Marital status			
Married or partnered	1,152 (88%)	901 (88%)	2,297 (89%)
Single	155 (12%)	126 (12%)	274 (11%)
Geographic remoteness			
Major city	1,148 (88%)	880 (86%)	2,291 (89%)
Inner/outer regional/remote	161 (12%)	147 (14%)	282 (11%)
Area of disadvantage^b^			
Least disadvantaged	732 (56%)	577 (56%)	1,414 (55%)
Most disadvantaged	577 (44%)	450 (44%)	1,157 (45%)
First pregnancy	552 (42%)	402 (39%)	1,033 (40%)

a TAFE, technical and further education. In this context, it refers to participants with a technical certificate as their highest level of education.

b Index of relative socio-economic disadvantage (33) was used to classify area of disadvantage.

### Aim 1: rate of change in the receipt of care post-implementation for all antenatal visits

**Figure 2:**
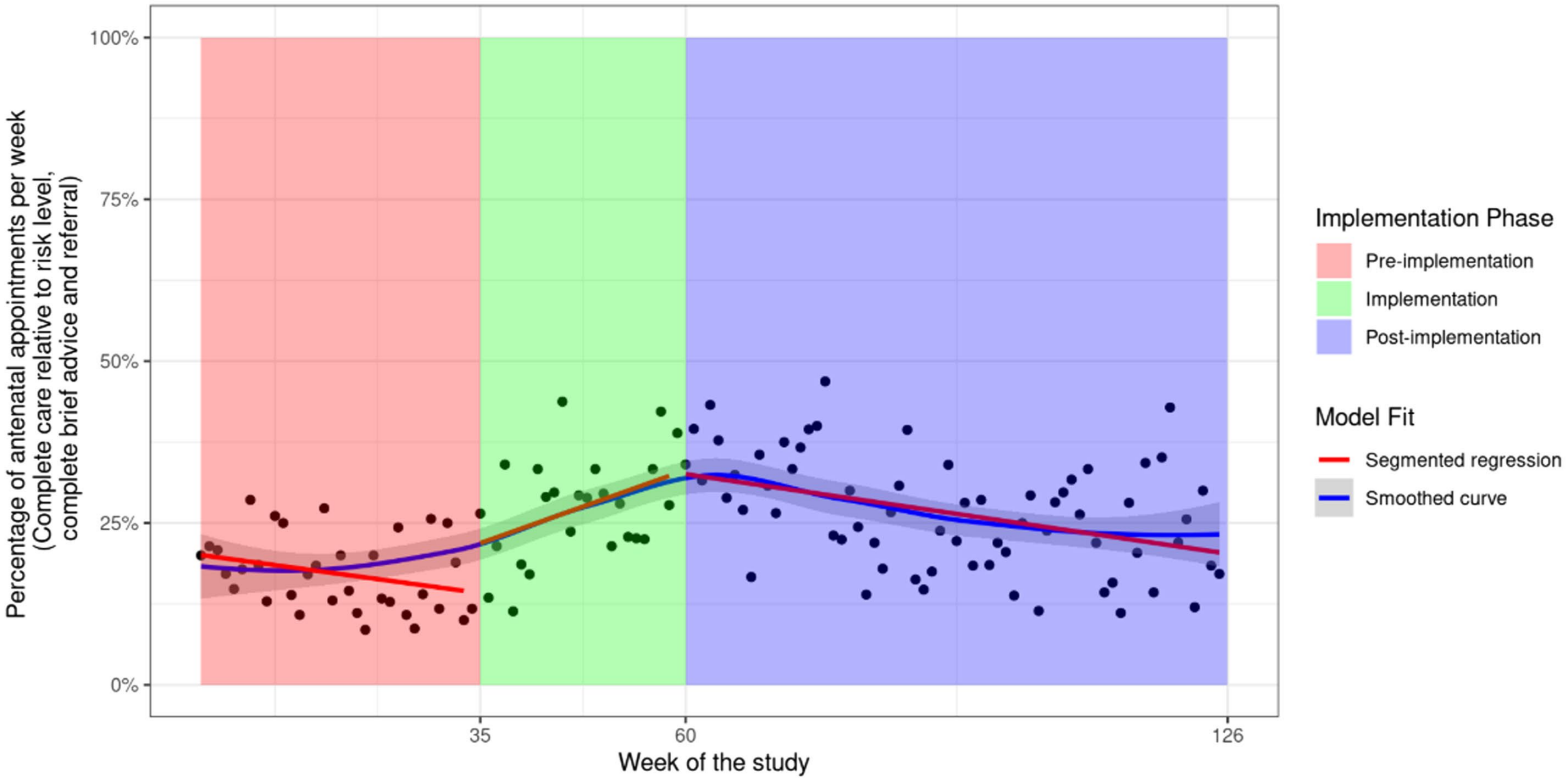
Fitted values of a linear segmented regression model including all antenatal appointments for outcome assessment of alcohol consumption and level of risk using the AUDIT-C and complete care relative to risk level. General Equation $Y_{t}=\alpha_{0}+\beta_{0}t+X_{1,t}\left(\alpha_{1}+\beta_{1}t\right)+X_{2,t}\left(\alpha_{2}+\beta_{2}t\right)$ . The two factor variables $X_{1,t}$ and $X_{2,t}$ are used in combination to indicate the pre-implementation ( $X_{1,t}=0,X_{2,t}=0\;)$ , implementation ( $X_{1,t}=1,X_{2,t}=0)$ , and post-implementation ( $X_{1,t}=0,X_{2,t}=1)$ periods. For each of these phases, the $\alpha_{i}$ represents intercepts or changes in intercept for the respective implementation phases, and $\beta_{i}$ represents the trend of the phase or the change in trend from the previous phase.

Table 2 presents the average weekly change in the proportion women, for all antenatal visits combined, reporting receipt of each element of guideline-recommended care during the post-implementation phase. Positive values represent an average weekly increase in the receipt of care, while negative values represent an average weekly decrease. A visual representation of the change for all components of guideline-recommended care appropriate to alcohol risk level (i.e., assessment and complete care) across all three phases of implementation (i.e., pre-implementation, implementation, and post-implementation) is shown in Figure 2 and for all other outcomes in the Supplementary File.

**Table 2:** Weekly change in the delivery of recommended care across the post-implementation phase for all antenatal visits combined and separately by initial visit and subsequent visits.

Outcome	Post-implementation % change per week (95% CI)		
	All visits combined	Initial visit	Subsequent visits
Assessment of alcohol consumption and level of risk using the AUDIT-C	−0.66 (−1.1, −0.26; *p* = 0.002)*	−0.20 (−0.86, 0.46; *p* = 0.5)	−0.97 (−1.4, −0.53; *p* < 0.001)*
Complete brief advice (safest not to consume and potential risks)	−0.63 (−1.1, −0.22; *p* = 0.003)*	−1.1 (−1.9, −0.33; *p* = 0.006)*	−0.50 (−1.00, −0.01; *p* = 0.044)*
Component one of brief advice: advice safest not to drink	−0.98 (−1.4, −0.52; *p* < 0.001)*	−0.96 (−1.7, −0.22; *p* = 0.011)*	−1.1 (−1.7, −0.49; *p* < 0.001)*
Component two of brief advice: advice on potential risks	−0.80 (−1.3, −0.28; *p* = 0.003)*	−1.2 (−2.0, −0.37; *p* = 0.005)*	−0.75 (−1.4, −0.09; *p* = 0.025)*
Complete care relative to risk level (complete brief advice and referral)	−0.64 (−1.1, −0.22; *p* = 0.003)*	−1.1 (−1.9, −0.31; *p* = 0.007)*	−0.51 (−0.99, −0.02; *p* = 0.040)*
Assessment of alcohol consumption and level of risk using the AUDIT-C and complete care relative to risk level	−0.36 (−0.72, 0.00; *p* = 0.050)	−0.40 (−0.76, −0.03; *p* = 0.034)*	−0.40 (−0.76, −0.03; *p* = 0.034)*

*Statistically significant rate of weekly change at *p* < 0.05.

Following an increase in recommended care during the implementation phase (Figure 2 and Supplementary File 1), all outcomes illustrate a significant decrease in the rate of care receipt during the post-implementation phase except for “assessment of alcohol and level of risk using the AUDIT-C and complete care relative to risk level,” which had a non-significant decrease with an average change of −0.36% (95%: −0.72, 0.00) per week (see Figure 2). For all other outcomes, the average weekly decrease in the receipt of care was statistically significant and ranged from −0.63% (95% CI: −1.10, −0.22) for “complete brief advice” to −1.00% (95% CI: −1.40, −0.52) for “advice safest not to drink” (see Table 2). A potential outlier in the post-implementation phase was observed for the outcome “assessment for alcohol consumption and level of risk using the AUDIT-C.” However, sensitivity analysis removing the outlier did not result in a meaningful difference in the results (−0.66% to −0.63% per week, see Supplementary File 1 Figure S2). As this outlier was not an error, it was not removed from the analysis.

### Aim 2: rate of change in the receipt of care post-implementation separately by antenatal visit

#### Initial visit only

Five of the six outcomes illustrated a significant decrease in the receipt of recommended care once initial implementation ended (see Table 2 and Supplementary File 2). There was minimal change in the rate to which women attending their initial visit received “assessment of alcohol consumption and level of risk using the AUDIT-C,” with a non-significant decrease observed for this outcome (Table 2 and Supplementary File 2 Figure S1).

#### Subsequent visits only

All outcomes illustrated a significant decrease in the receipt of recommended care once initial implementation ended (see Table 2 and Supplementary File 3).

### Aim 3: identify specific points during post-implementation where the rate of change in the receipt of care is more rapid

The results from the break-point analysis are illustrated in Supplementary File 4. All outcomes except “assessment of alcohol consumption and level of risk using the AUDIT-C,” illustrated an immediate and rapid decline in the receipt of care post-implementation, until approximately 30 weeks post-implementation. From 30 weeks post-implementation, the rates of care appear to stabilize. The outcome “assessment of alcohol consumption and level of risk using the AUDIT-C” illustrates an immediate decline until approximately 60 weeks when rates begin to stabilize (Supplementary File 4 Figure S1). However, when a potential outlier for this outcome is removed, the break-point analysis suggests an immediate and rapid decline until approximately 9 weeks post-implementation (Supplementary File 4 Figure S2).

### Aim 4: the extent of care receipt at the end of each implementation phase and directly following key time points from the break-point analysis

Table 3 presents the percentage of women receiving care for the last 4 weeks of each implementation phase (i.e., pre-implementation, implementation, and post-implementation) and for the 4 weeks following 30 weeks post-implementation as this was identified as a significant break point for all but one outcome. The percentage of women receiving recommended care was low at pre-implementation, then increased during active implementation, and then reduced during post-implementation. However, the percentage of women receiving recommended care post-implementation never reduced to pre-implementation levels for any of the outcomes.

**Table 3:** Percentage of women reporting receipt of recommended care elements for the last 4 weeks in each implementation phase and at 30 weeks post-implementation for all antenatal visits combined.

Outcome	Percentage of women reporting receipt of each recommended care elements			
	Pre-implementation (*n* = 178 women)	Implementation (*n* = 161 women)	30 weeks post-implementation (*n* = 177 women)	End of post-implementation (*n* = 126 women)
Assessment of alcohol consumption and level of risk using the AUDIT-C	27% (*n* = 48)	45% (*n* = 73)	44% (*n* = 77)	37% (*n* = 46)
Complete brief advice (safest not to consume and potential risks)	16% (*n* = 29)	35% (*n* = 57)	25% (*n* = 44)	22% (*n* = 28)
Component one of brief advice: advice safest not to drink	32% (*n* = 57)	56% (*n* = 90)	45% (*n* = 79)	41% (*n* = 52)
Component two of brief advice: advice on potential risks	21% (*n* = 37)	41% (*n* = 67)	31% (*n* = 54)	29% (*n* = 36)
Complete care relative to risk level (complete brief advice and referral)	16% (*n* = 28)	35% (*n* = 57)	25% (*n* = 44)	22% (*n* = 28)
Assessment of alcohol consumption and level of risk using the AUDIT-C and complete care relative to risk level	11% (*n* = 19)	25% (*n* = 41)	16% (*n* = 29)	17% (*n* = 22)

## Discussion

This study used existing data from a stepped-wedge randomized controlled trial to explore the change in recommended antenatal care delivery following an effective implementation intervention. It overcomes existing limitations of the field by using an appropriate analytic approach to explore changes in outcomes across the implementation process, using recently collected data (14). It helps to identify if and when the implementation intervention effects may reduce (“wash out”), and when additional support may be required. Encouragingly, rates of care receipt were positively influenced by the implementation intervention (6). However, rates for all outcomes declined after the implementation phase ended, with immediate and more rapid declines appearing to occur for all but one outcome until approximately 30 weeks following the completion of implementation support. For most outcomes, the prevalence of care seemed to stabilize from this point, although longer follow-up assessment is needed to confirm if further reductions occur.

These findings illustrate an immediate decline in women’s receipt of recommended care once implementation support ended, regardless of what antenatal visit women were attending, and despite the intervention including a number of implementation strategies that were ongoing through integration with existing resources and systems, such as those that were believed to support sustainment due to their system-level technological changes, including reminders built into the existing medical record system. However, for two of the 18 outcomes, this decline was not statistically significant, one of which was for outcomes assessed in women attending their initial visit. This is not surprising as the rates of recommended care delivered prior to the implementation intervention were higher for women attending their initial visit compared to those attending a subsequent visit (6). Furthermore, the implementation intervention was found to have a greater effect on outcomes for women attending their subsequent visits than those attending their initial visit (6); thus, there was the potential for a greater level of implementation effect to be lost for women attending subsequent visits as rates of recommended care were already high for initial visits. The findings are consistent with systematic reviews and individual trials that have identified a decline in implementation following the withdrawal of funding or completion of implementation support (8, 9, 15, 26). The findings highlight the difficulties in sustaining long-term changes in clinical care, which are likely impacted by a range of organizational and outer environmental factors that can change rapidly over time (e.g., staffing, policies, regulations, and evidence) (27, 28). As recommended, ongoing monitoring and adaptation of the intervention and implementation support may be needed to ensure that successful practice changes continue to fit and are integrated within changing clinical environments (27). In this specific instance, additional support may need to be considered within 30 weeks following implementation.

It is likely that an array of contextual factors impact the declines observed in this study. However, our understanding of the clinical setting and existing evidence suggests that two possible factors may have had an impact on the sustainment of recommended care being delivered. First, a lack of capacity or capability for clinicians to provide recommended care due to the withdrawal of the specifically trained clinical champion who educated and supported clinicians during this trial, and the provision of academic detailing and audit and feedback, with evidence suggesting that clinical champions in particular are influential to sustainment (10, 17, 29). The second relates to reductions in the number of staff who had been exposed to the initial implementation intervention due to regular clinical rotations and workforce turnover. Post-implementation surveys with staff 12 weeks after the intervention found that only approximately 70% had received the training. The high rotation of staff from antenatal clinics may reduce the impact of a number of the implementation strategies, including educational meetings, academic detailing, and support from clinical champions (30). Additional strategies, such as booster education for existing staff and inclusion of training in the orientation of new staff, may be needed to ensure sustainment. Working with services to develop education and training that can be incorporated, as much as possible, into current systems and workflows will be necessary to ensure that such strategies can be embedded into routine practice. We are currently testing the impact and feasibility of delivering such sustainment strategies in the maternal health setting (31).

Limited research has been conducted on sustainability-specific strategies to address declines following effective implementation support. We are aware of one systematic review that assessed sustainability strategies, which focused on public health interventions (32). Only six of the 26 included studies reported the use of strategies specifically to support sustainment (32). The effect of such strategies was not assessed, providing limited knowledge as to which strategies maybe most effective. Further research is required that involves the development and conduct of specific sustainability trials aimed at supporting the long-term receipt of recommended care generally and addressing pregnant women’s consumption of alcohol specifically (31).

### Limitations

This is a secondary analysis. The design and sample size were not developed *a priori* to undertake this study. Consequently, the findings should be interpreted as hypothesis and concept generating only. Only one of the three sectors from the primary trial was included, due to the small sample sizes within the other two sectors. Finally, we only assessed implementation rates until 17 months post-implementation, which is contrary to recommendations that sustainment should be considered from 2 years post-implementation. However, our finding that care delivery reduced by the end of the 17-month post-implementation period highlights the need to consider issues relating to sustainment earlier in the implementation continuum.

## Conclusion

This secondary analysis provided an opportunity to explore the rate, time points, and extent of change in women’s receipt of recommended antenatal care addressing alcohol consumption during pregnancy, following the withdrawal of effective implementation support. Consistent with previous research, we found that the effects of the implementation intervention declined after active implementation were completed. However, for most outcomes, this decline appears to stabilize from approximately 30 weeks post-implementation. The results suggest the potential need for additional sustainability strategies initially after the withdrawal of implementation support to ensure that the benefits of delivering guideline-recommended care are continued long term.

## Data Availability

The datasets presented in this article are not readily available because data is housed with the authors following ethical approval. Requests to access the datasets should be directed to emma.doherty@health.nsw.gov.au.

## Author’s note

The author, Belinda Tully is from Gomeroi Nation.

## References

[1] Oei JL. Alcohol use in pregnancy and its impact on the mother and child. Addiction. (2020) 115:2148–63. doi: 10.1111/add.15036, PMID: 32149441

[2] Australian Government Department of Health. Clinical practice guidelines: pregnancy care. Canberra: Australian Government Department of Health (2020).

[3] Graves L Carson G Poole N Patel T Bigalky J Green CR. Guideline no. 405: screening and counselling for alcohol consumption during pregnancy. J Obstet Gynaecol Can. (2020) 42:1158–73. doi: 10.1016/j.jogc.2020.03.002 32900457

[4] World Health Organisation. Guidelines for the identification and management of substance use and substance use disorders in pregnancy. Geneva: World Health Organisation (2014). 24783312

[5] Doherty E Kingsland M Wiggers J Anderson AE Elliott EJ Symonds I. Barriers to the implementation of clinical guidelines for maternal alcohol consumption in antenatal services: a survey using the theoretical domains framework. Health Promot J Austr. (2020) 31:133–9. doi: 10.1002/hpja.258, PMID: 31087792

[6] Doherty E Kingsland M Elliott EJ Tully B Wolfenden L Dunlop A. Practice change intervention to improve antenatal care addressing alcohol consumption during pregnancy: a randomised stepped-wedge controlled trial. BMC Pregnancy Childbirth. (2022) 22:1–17. doi: 10.1186/s12884-022-04646-7, PMID: 35448996 PMC9027411

[7] Moullin JC Sklar M Green A Dickson KS Stadnick NA Reeder K. Advancing the pragmatic measurement of sustainment: a narrative review of measures. Implement Sci Commun. (2020) 1:76. doi: 10.1186/s43058-020-00068-8, PMID: 32964208 PMC7499830

[8] Ament SM de Groot JJ Maessen JM Dirksen CD van der Weijden T Kleijnen J. Sustainability of professionals’ adherence to clinical practice guidelines in medical care: a systematic review. BMJ Open. (2015) 5:e008073. doi: 10.1136/bmjopen-2015-008073, PMID: 26715477 PMC4710818

[9] Herlitz L MacIntyre H Osborn T Bonell C. The sustainability of public health interventions in schools: a systematic review. Implement Sci. (2020) 15:1–28. doi: 10.1186/s13012-019-0961-8 31906983 PMC6945701

[10] Wiltsey Stirman S Kimberly J Cook N Calloway A Castro F Charns M. The sustainability of new programs and innovations: a review of the empirical literature and recommendations for future research. Implement Sci. (2012) 7:1–19. doi: 10.1186/1748-5908-7-17 PMC3317864 22417162

[11] Wong DR Schaper H Saldana L. Rates of sustainment in the universal stages of implementation completion. Implement Sci Commun. (2022) 5:2. doi: 10.1186/s43058-021-00250-6 PMC8727078 34983685

[12] Moore JE Mascarenhas A Bain J Straus S. Developing a comprehensive definition of sustainability. Implement Sci. (2017) 12:110. doi: 10.1186/s13012-017-0637-1 28865479 PMC5581411

[13] Chambers DA Glasgow RE Stange KC. The dynamic sustainability framework: addressing the paradox of sustainment amid ongoing change. Implement Sci. (2013) 8:1–11. doi: 10.1186/1748-5908-8-11 24088228 PMC3852739

[14] Proctor E Luke D Calhoun A McMillen C Brownson R McCrary S. Sustainability of evidence-based healthcare: research agenda, methodological advances, and infrastructure support. Implement Sci. (2015) 10:88. doi: 10.1186/s13012-015-0274-5, PMID: 26062907 PMC4494699

[15] Levi CR Attia JA D'Este C Ryan AE Henskens F Kerr E. Cluster-randomized trial of thrombolysis implementation support in metropolitan and regional Australian stroke centers: lessons for individual and systems behavior change. J Am Heart Assoc. (2020) 9:e012732. doi: 10.1161/JAHA.119.012732, PMID: 31973599 PMC7033885

[16] MacBride-Stewart S Marwick C Houston N Watt I Patton A Guthrie B. Evaluation of a complex intervention to improve primary care prescribing: a phase IV segmented regression interrupted time series analysis. Br J Gen Pract. (2017) 67:e352–60. doi: 10.3399/bjgp17X690437 28347986 PMC5409419

[17] Singh M Gmyrek A Hernandez A Damon D Hayashi S. Sustaining screening, brief intervention and referral to treatment (SBIRT) services in health-care settings. Addiction. (2017) 112:92–100. doi: 10.1111/add.13654, PMID: 28074565

[18] King DK Ondersma SJ McRee BG German JS Loree AM Harlowe A. Using planned and unplanned adaptation to implement universal alcohol screening and brief intervention to prevent alcoho-exposed pregnancies in four primary care health systems. Subst Use Addctn J. (2025) 46:439–51. doi: 10.1177/29767342241271404, PMID: 39305032 PMC11910335

[19] Hemming K Haines TP Chilton PJ Girling AJ Lilford RJ. The stepped wedge cluster randomised trial: rationale, design, analysis, and reporting. BMJ. (2015) 350:h391. doi: 10.1136/bmj.h391, PMID: 25662947

[20] Kingsland M Doherty E Anderson AE Crooks K Tully B Tremain D. A practice change intervention to improve antenatal care addressing alcohol consumption by women during pregnancy: research protocol for a randomised stepped-wedge cluster trial. Implement Sci. (2018) 13:1–14. doi: 10.1186/s13012-018-0806-x 30126437 PMC6102816

[21] Bradley KA Bush KR Epler AJ Dobie DJ Davis TM Sporleder JL. Two brief alcohol-screening tests from the alcohol use disorders identification test (AUDIT): validation in a female veterans affairs patient population. Arch Intern Med. (2003) 163:821–9. doi: 10.1001/archinte.163.7.821, PMID: 12695273

[22] Bush K Kivlahan DR McDonell MB Fihn SD Bradley KA. The AUDIT alcohol consumption questions (AUDIT-C): an effective brief screening test for problem drinking. Ambulatory care quality improvement project (ACQUIP). Arch Intern Med. (1998) 158:1789–95. doi: 10.1001/archinte.158.16.1789, PMID: 9738608

[23] New South Wales Government. (2022). Get healthy: information and coaching service. Available online at: . (https://www.gethealthynsw.com.au/program/get-healthy-in-pregnancy/)

[24] Foundation for Alcohol Research and Education. (2018). Information for health professionals on assessing alcohol consumption in pregnancy using AUDIT-C Australian Department of Health. Available online at: . (http://www.alcohol.gov.au/internet/alcohol/publishing.nsf/Content/wwtk-audit-c)

[25] R Core Team. R: A language and environment for statistical computing. Vienna, Austria: R Foundation for Statistical Computing (2022).

[26] Williams L Daggett V Slaven JE Yu Z Sager D Myers J. A cluster-randomised quality improvement study to improve two inpatient stroke quality indicators. BMJ Qual Saf. (2016) 25:257–64. doi: 10.1136/bmjqs-2015-004188, PMID: 26303644

[27] Chambers DA. Advancing sustainability research: challenging existing paradigms. J Public Health Dent. (2011) 71:S99–S100. doi: 10.1111/j.1752-7325.2011.00238.x, PMID: 21656965

[28] Shelton RC Cooper BR Stirman SW. The sustainability of evidence-based interventions and practices in public health and health care. Annu Rev Public Health. (2018) 39:55–76. doi: 10.1146/annurev-publhealth-040617-014731, PMID: 29328872

[29] Blasinsky M Goldman HH Unützer J. Project IMPACT: a report on barriers and facilitators to sustainability. Adm Policy Ment Health Ment Health Serv Res. (2006) 33:718–29. doi: 10.1007/s10488-006-0086-7, PMID: 16967339

[30] Dray J Licata M Doherty E Tully B Williams B Curtin S. Enhancing clinician participation in quality improvement training: implementation and impact of an evidence-based initiative to maximise antenatal clinician participation in training regarding women’s alcohol consumption during pregnancy. BMC Health Serv Res. (2022) 22:402. doi: 10.1186/s12913-022-07717-9 35351113 PMC8962084

[31] Doherty E Wiggers J Nathan J Hall A Wolfenden L Tully B. Iterative delivery of an implementation support package to increase and sustain the routine provision of antenatal care addressing alcohol consumption during pregnancy: study protocol for a steppedwedge cluster trial. BMJ Open. (2022) 12:e063486. doi: 10.1136/bmjopen-2022-063486, PMID: 35882461 PMC9330336

[32] Hailemariam M Bustos T Montgomery B Barajas R Evans LB Drahota A. Evidence-based intervention sustainability strategies: a systematic review. Implement Sci. (2019) 14:57. doi: 10.1186/s13012-019-0910-6, PMID: 31171004 PMC6554955

[33] Australian Bureau of Statistics. SEIFA: socio-economic indexes for areas. Canberra: Australian Government (2008).

