# Supplementary material for: Longitudinal exploration of the delivery of care following a successful antenatal practice change intervention
Source: Front Med (Lausanne). 2025 May 9;12:1476083. doi: 10.3389/fmed.2025.1476083 (PMC12098108; doi:10.3389/fmed.2025.1476083)
Supplement: Supplementary file 1 [file Supplementary_file_1.docx]

**Supplementary File 1: Fitted values of linear segmented regressions models including all antenatal visits**


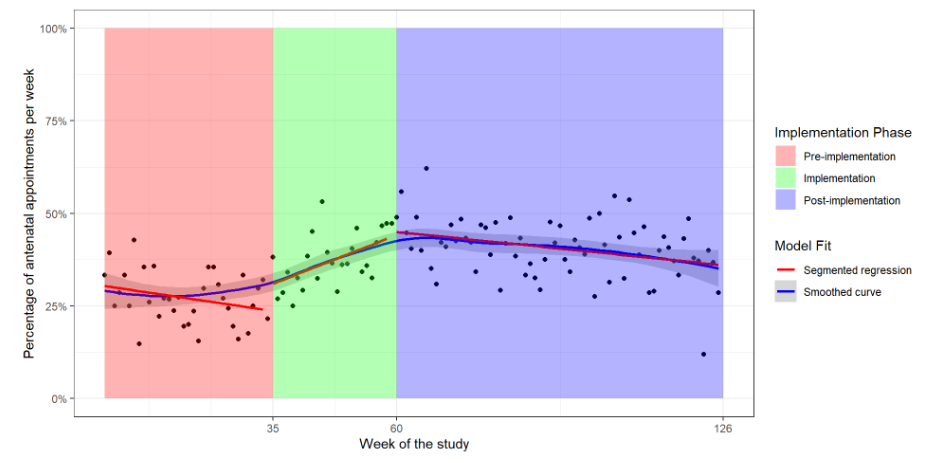


Figure S1: Fitted values of a linear segmented regression model including all antenatal appointments for outcome assessment for alcohol consumption and level of risk using the AUDIT-C


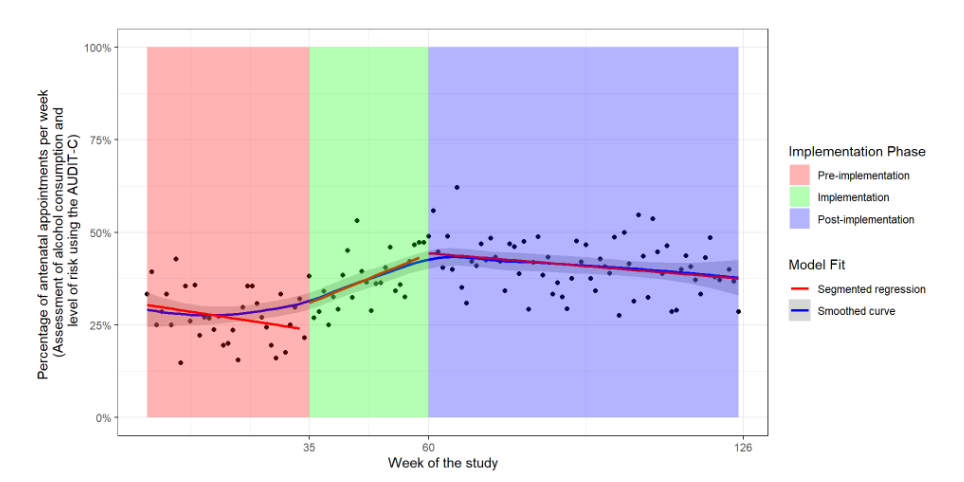


Figure S2: Fitted values of a linear segmented regression model including all antenatal appointments for outcome assessment for alcohol consumption and level of risk using the AUDIT-C excluding outlier


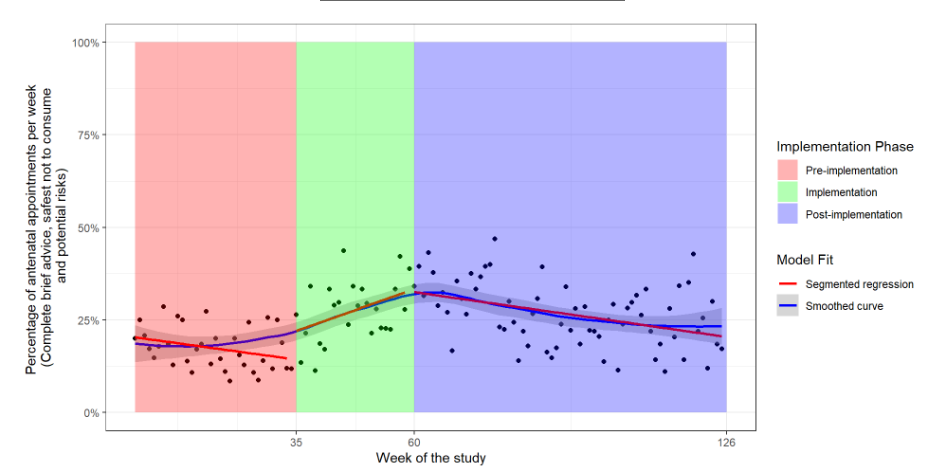


Figure S3. Fitted values of a linear segmented regression model including all antenatal appointments for outcome brief advice regarding the risks of consuming alcohol during pregnancy


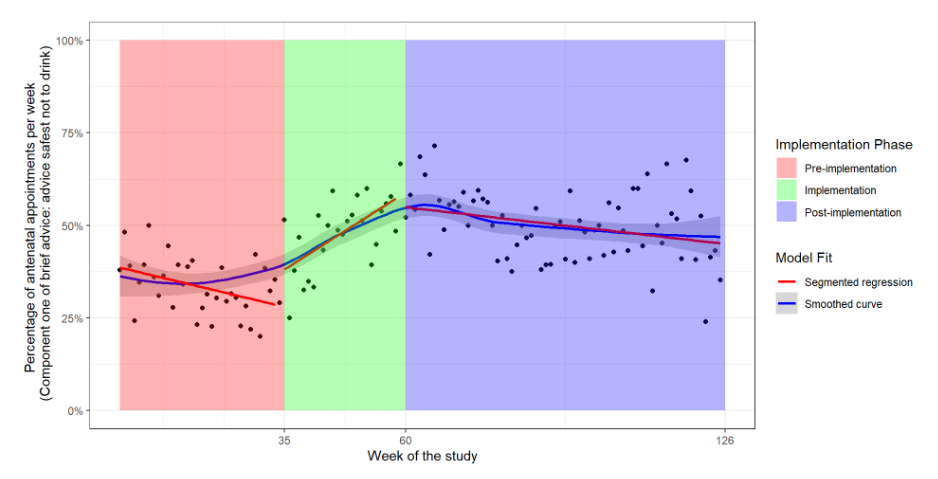


Figure S4. Fitted values of a linear segmented regression model including all antenatal appointments for outcome component of brief advice: advice not to drink


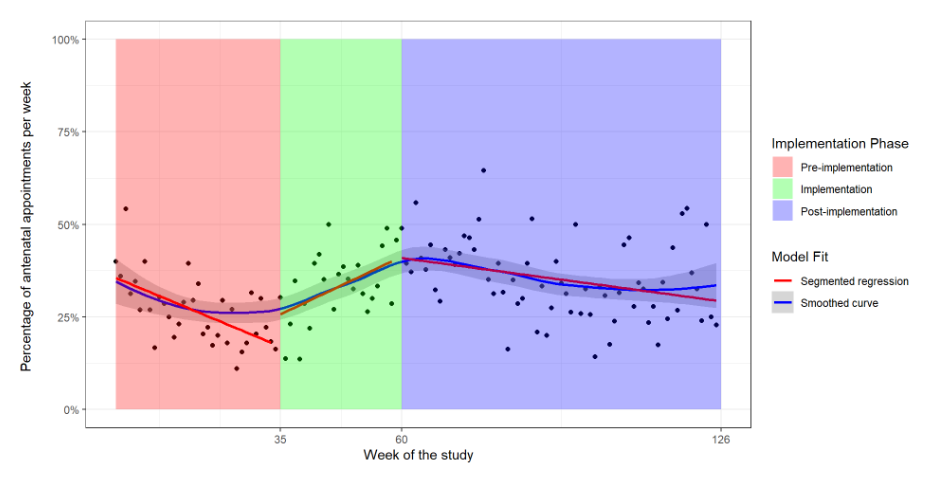


Figure S5. Fitted values of a linear segmented regression model including all antenatal appointments for outcome component of brief advice: advice on potential risks


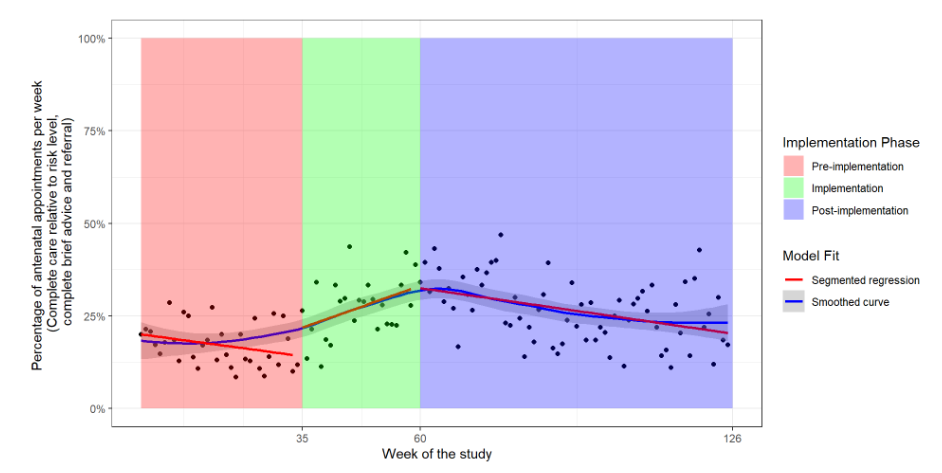


Figure S6. Fitted values of a linear segmented regression model including all antenatal appointments for outcome complete care relative to risk level (complete brief advice and referral)

**Supplementary File 2: Fitted values of linear segmented regressions models excluding women attending their subsequent antenatal visit**


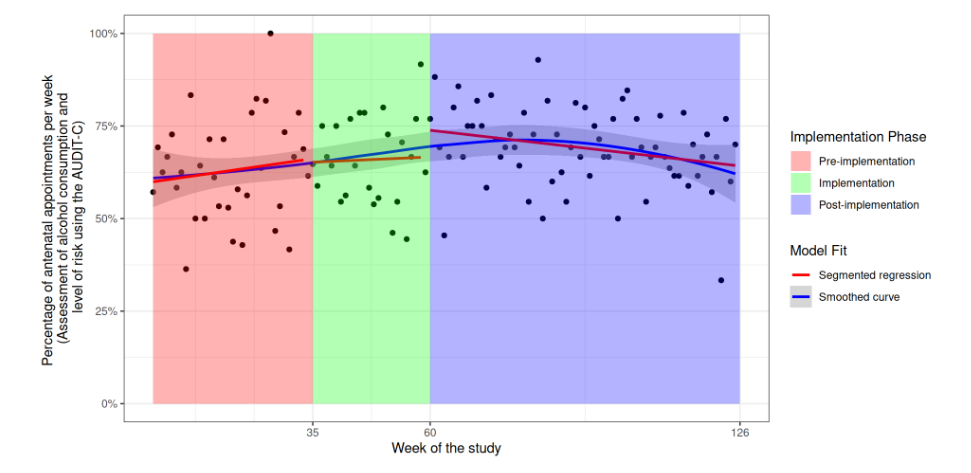


Figure S1: Fitted values of a linear segmented regression model excluding subsequent antenatal appointments for outcome assessment for alcohol consumption and level of risk using the AUDIT-C


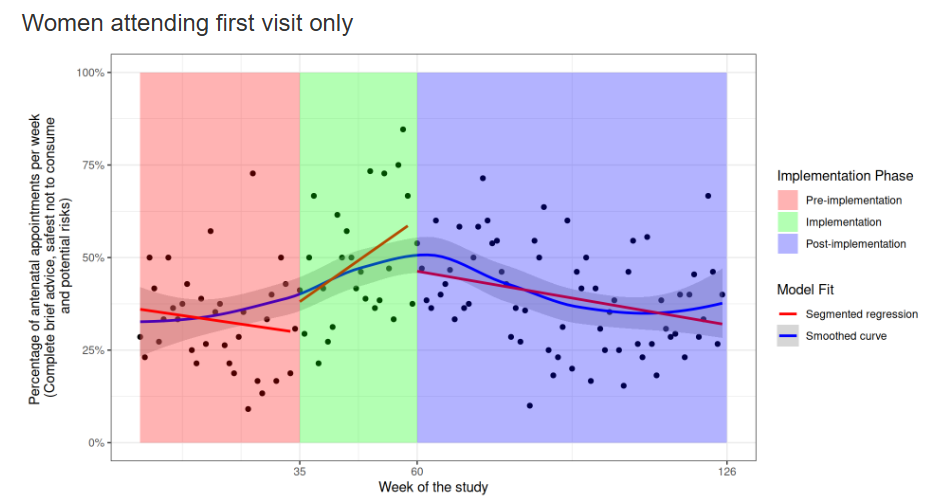


Figure S2: Fitted values of a linear segmented regression model excluding subsequent antenatal appointments for outcome complete brief advice (safest not to consume and potential risks)


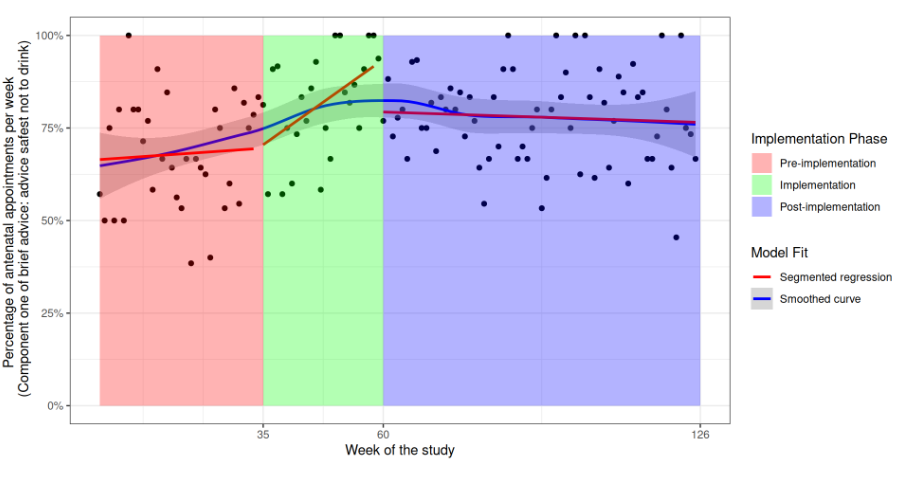


Figure S3. Fitted values of a linear segmented regression model excluding subsequent antenatal appointments for outcome component of brief advice: advice not to drink


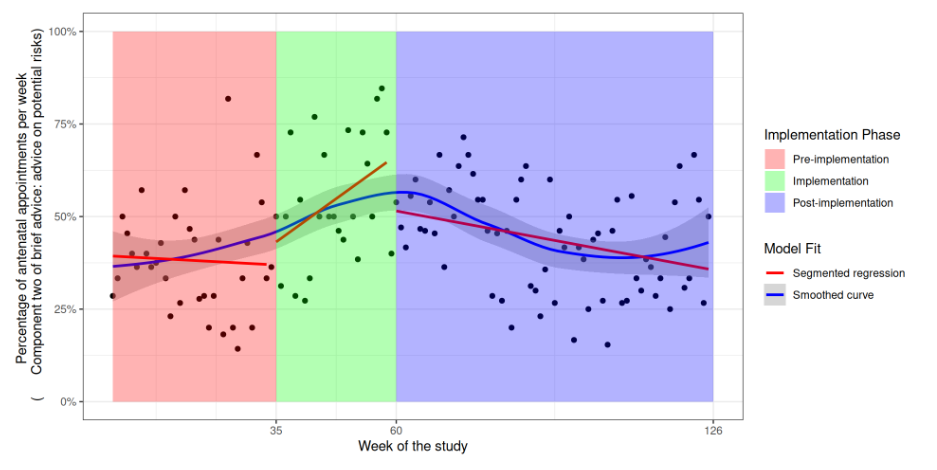


Figure S4. Fitted values of a linear segmented regression model excluding subsequent antenatal appointments for outcome component of brief advice: advice on potential risks


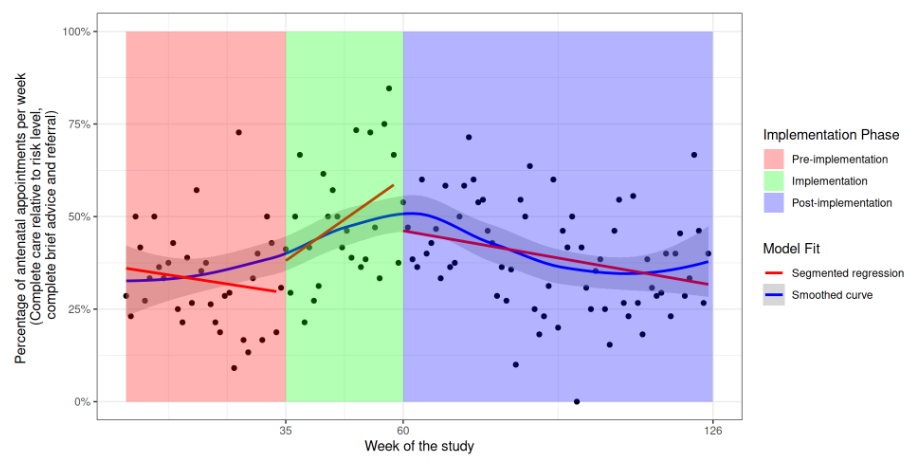


Figure S5. Fitted values of a linear segmented regression model excluding subsequent antenatal appointments for outcome complete care relative to risk level (complete brief advice and referral)


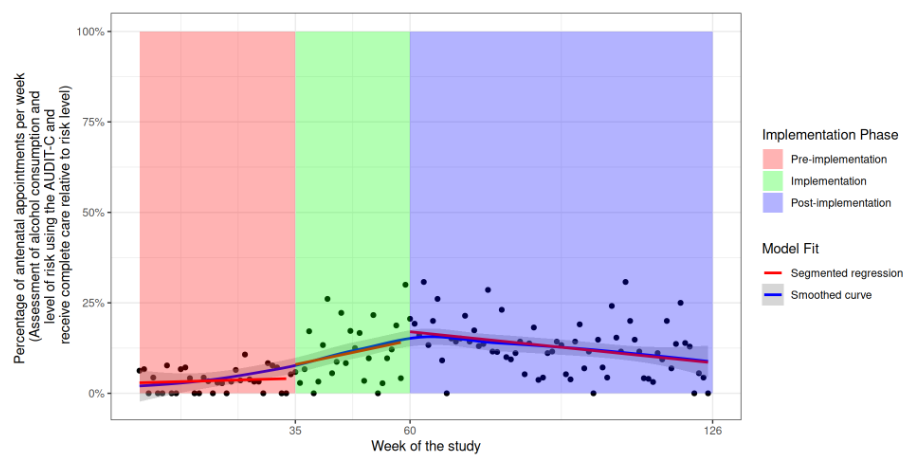


Figure S6. Fitted values of a linear segmented regression model excluding subsequent antenatal appointments for outcome assessment of alcohol consumption and level of risk using the AUDIT-C and complete care relative to risk level

**Supplementary File 3: Fitted values of linear segmented regressions models excluding women attending their initial antenatal visit**


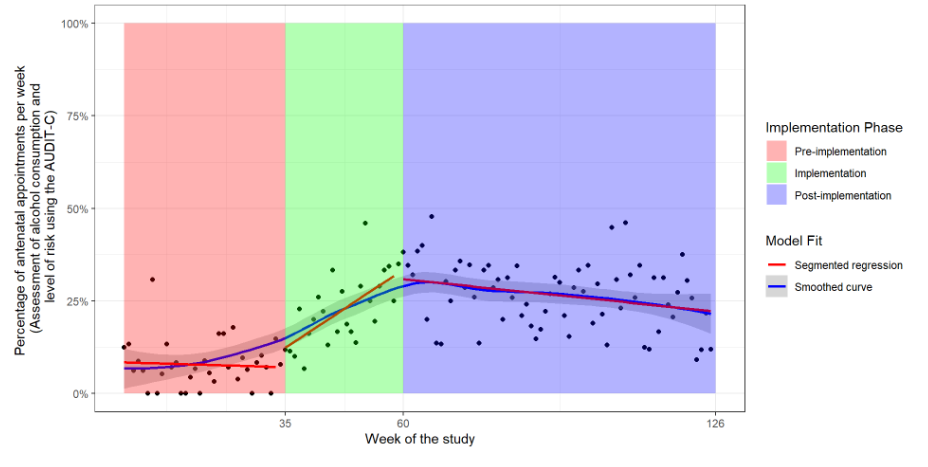


Figure S1: Fitted values of a linear segmented regression model excluding initial antenatal appointments for outcome assessment for alcohol consumption and level of risk using the AUDIT-C


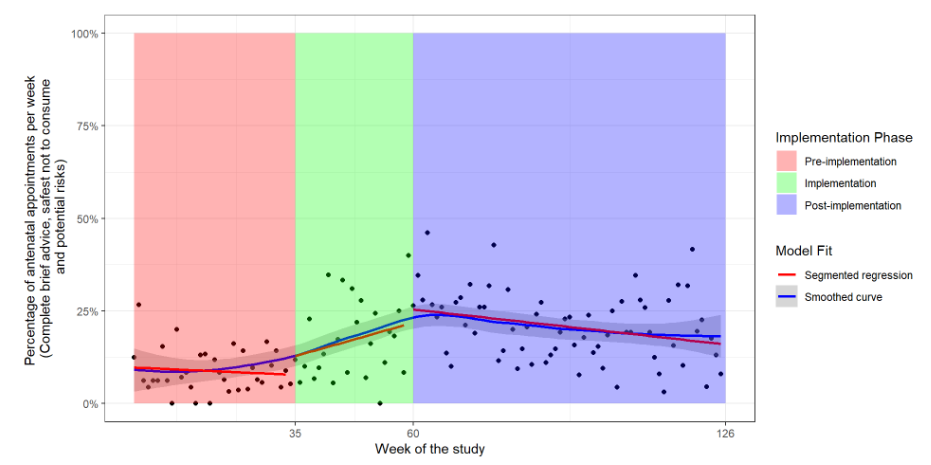


Figure S2: Fitted values of a linear segmented regression model excluding initial antenatal appointments for outcome complete brief advice (safest not to consume and potential risks)


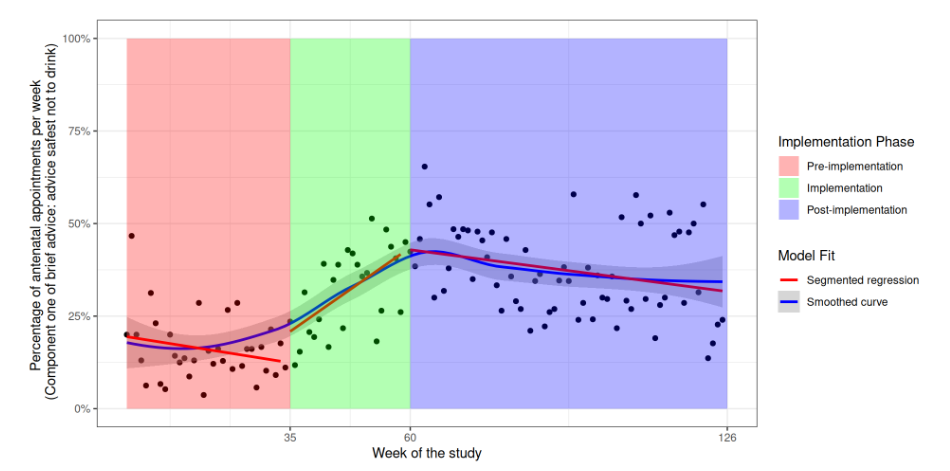


Figure S3. Fitted values of a linear segmented regression model excluding initial antenatal appointments for outcome component of brief advice: advice not to drink


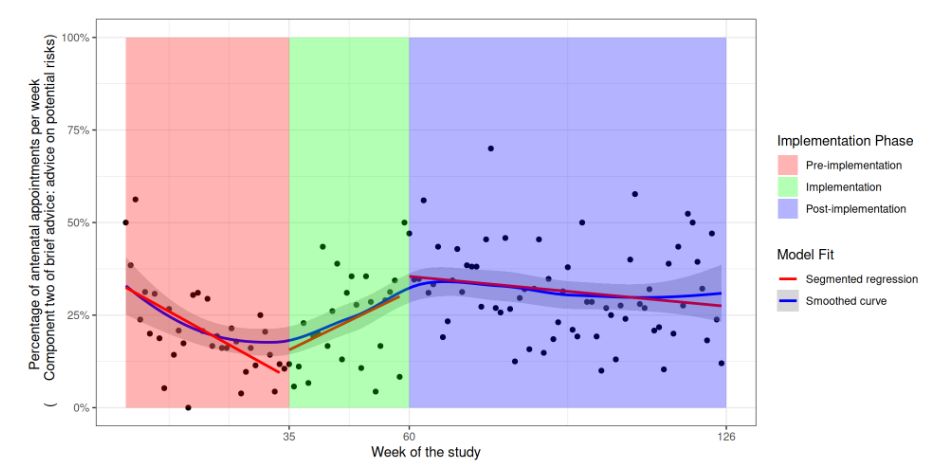


Figure S4. Fitted values of a linear segmented regression model excluding initial antenatal appointments for outcome component of brief advice: advice on potential risks


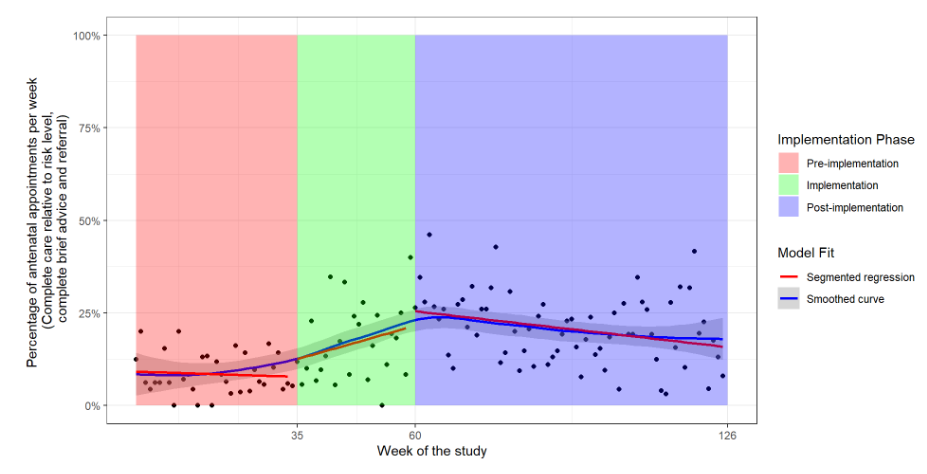


Figure S5. Fitted values of a linear segmented regression model excluding initial antenatal appointments for outcome complete care relative to risk level (complete brief advice and referral)


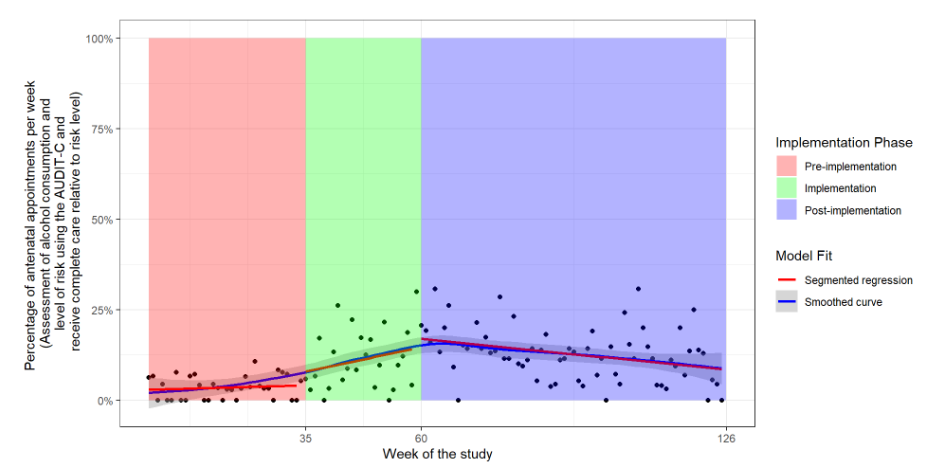


Figure S6. Fitted values of a linear segmented regression model excluding initial antenatal appointments for outcome assessment of alcohol consumption and level of risk using the AUDIT-C and complete care relative to risk level

**Supplementary File 4: Fitted values of linear segmented regressions models from break-point analysis including all antenatal visits**


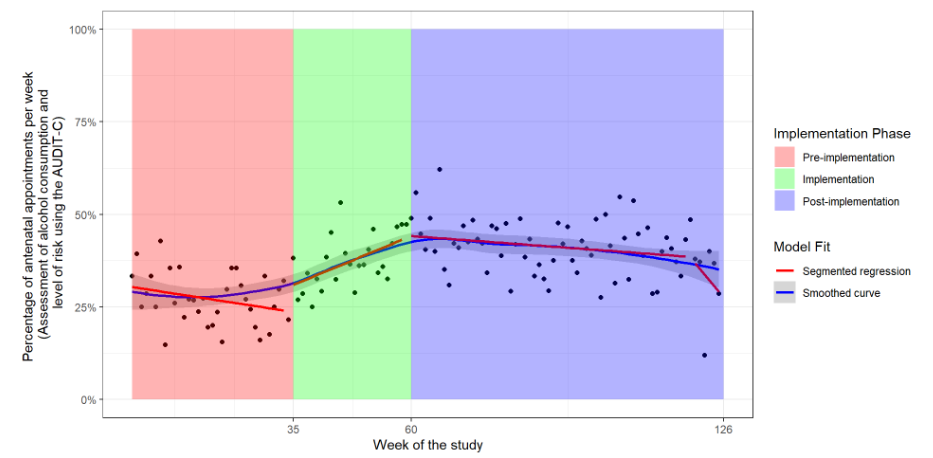


Figure S1: Fitted values of break-point analysis including all antenatal appointments for outcome assessment for alcohol consumption and level of risk using the AUDIT-C


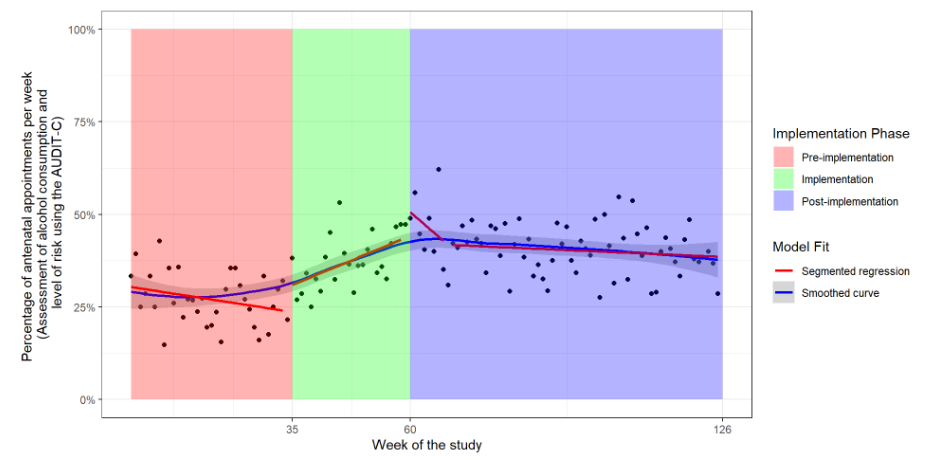


Figure S2: Fitted values of break-point analysis including all antenatal appointments for outcome assessment for alcohol consumption and level of risk using the AUDIT-C excluding outlier


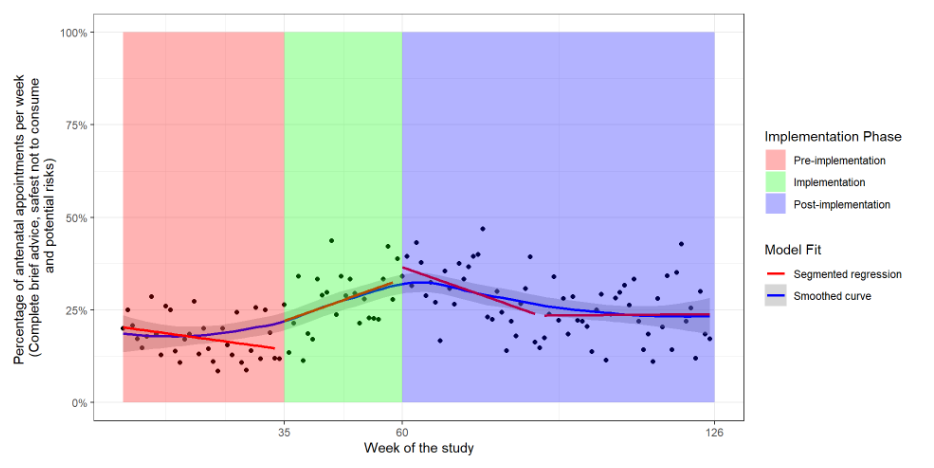


Figure S3. Fitted values of break-point analysis including all antenatal appointments for outcome brief advice regarding the risks of consuming alcohol during pregnancy


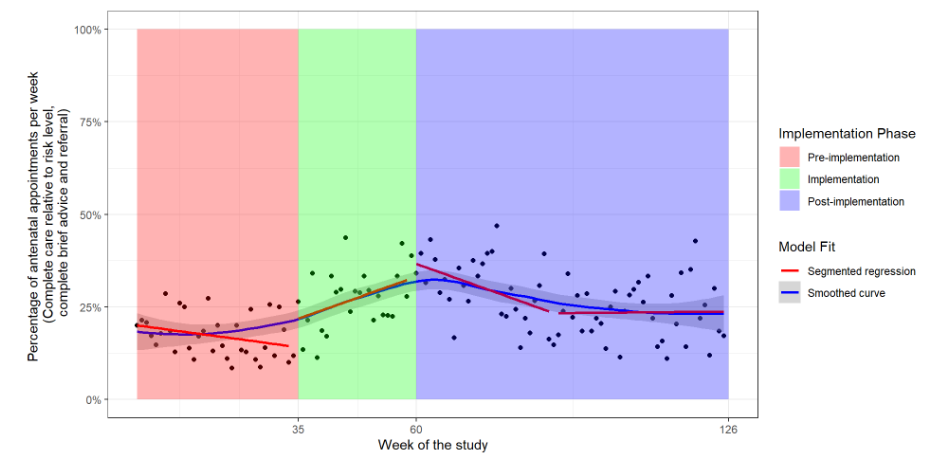


Figure S4. Fitted values of break-point analysis including all antenatal appointments for outcome complete care relative to risk level (complete brief advice and referral)


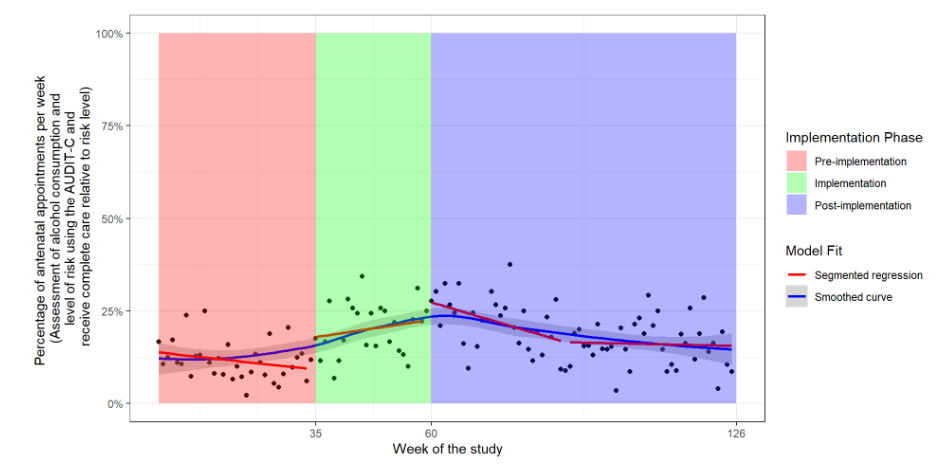


Figure S5. Fitted values of break-point analysis including all antenatal appointments for outcome assessment of alcohol consumption and level of risk using the AUDIT-C and complete care relative to risk level


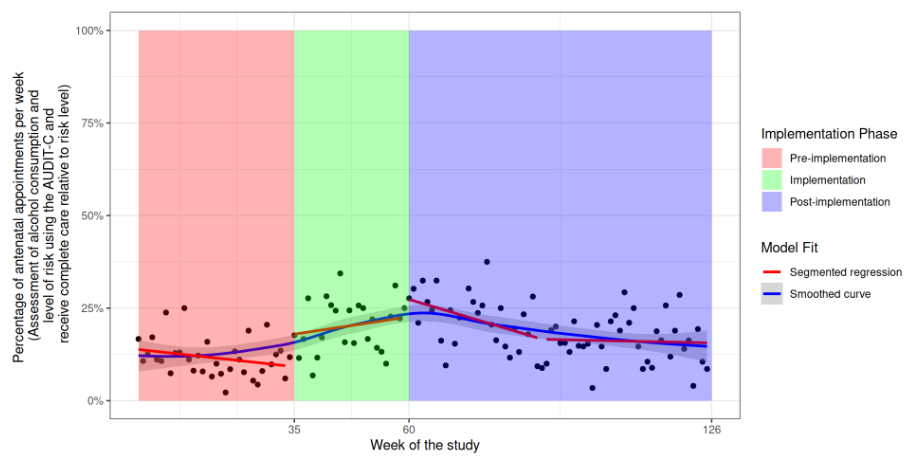


Figure S6. Fitted values of break-point analysis including all antenatal appointments for outcome assessment of alcohol consumption and level of risk using the AUDIT-C and complete care relative to risk level
